# Future perspectives of heart rate and oxygenation monitoring in the neonatal intensive care unit – a narrative review

**DOI:** 10.1007/s10877-025-01310-1

**Published:** 2025-06-27

**Authors:** Emma Williams, Rudolf Ascherl, Vincent D. Gaertner, Greta Sibrecht, Serife Kurul, Marie-Louise Herrmann, Eniko Szakmar, Genny Raffaeli, Ilia Bresesti, Kerstin Jost

**Affiliations:** 1 Department of Women and Children’s Health, Faculty of Life Sciences & Medicine, King’s College London, London, UK; 2 Department of Neonatology, Hospital for Children and Adolescents, University of Leipzig Medical Center, Leipzig, Germany; 3 Division of Neonatology, Dr von Hauner Children’s Hospital, Ludwig-Maximilians- Universität München, Munich, Germany; 4 II Department of Neonatology, Poznan University of Medical Sciences, Poznan, Poland; 5 Department of Neonatal and Pediatric Intensive Care, Division of Neonatology, Erasmus Medical Center - Sophia Children’s Hospital, Rotterdam, The Netherlands; 6 Department of Neonatology, University Hospital Leipzig, Leipzig, Germany; 7 Division of Neonatology, Pediatric Center, Semmelweis University, Budapest, Hungary; 8 Fondazione IRCCS Ca’ Granda Ospedale Maggiore Policlinico, NICU, Milan, Italy; 9 Department of Clinical Sciences and Community Health, University of Milan, Milan, Italy; 10 Division of Neonatology, ASST – Settelaghi, “F. Del Ponte” Hospital, University of Insubria, Varese, Italy; 11 Department of Neonatology, University Children´s Hospital Basel, Basel, Switzerland; 12 Department of Women´s and Children´s Health, Karolinska Institute, Stockholm, Sweden

**Keywords:** Heart rate, Monitoring, Neonatal, Oxygen, Technology

## Abstract

**Purpose:**

Vital sign monitoring plays a pivotal role in assessing and managing the clinical condition of vulnerable newborn infants in the delivery room and in the neonatal intensive care unit (NICU), with advancements in technology over the last years paving the way for newer and less invasive monitoring techniques.

**Methods:**

We conducted a narrative review of the literature in PubMed, Embase, GoogleScholar, and ClinicalTrials.gov. to describe newer technologies in neonatal monitoring of heart rate and oxygen saturation including secondary data-use, focusing also on promising studies which are currently underway.

**Results:**

Innovations such as photoplethysmography, wireless skin sensors, spectroscopy and tremolo sonification can provide a continuous and comprehensive assessment of neonatal vital sign monitoring, including heart rate and oxygen saturations, allowing for the enhancement of early detection of potential complications. Moreover advanced mathematical models, such as heart rate characteristic variability and closed loop automated systems, have shown promise in processing and storing vast amounts of data, aiding in the early prediction of adverse clinical outcomes, supporting decision-making and guiding the development of future studies.

**Conclusion:**

As the field of vital sign monitoring in the NICU continues to evolve, it is essential to address challenges related to novel modalities, data privacy, algorithm accuracy, and seamless integration into existing healthcare systems. By harnessing the potential of innovative technologies, the future of vital sign monitoring in the NICU promises improved neonatal outcomes, enhanced healthcare delivery and facilitation of individualisation of care.

## Impact

Heart rate and oxygen saturation are essential vital signs that are closely monitored in the neonatal intensive care unit.

Different approaches in either new measuring devices or combination of existing data in the form of calculated values are tested to better understand physiological and pathological vital sign behaviour.

This review highlights the potential benefit of new modalities as well as the need for integration into clinical practice to overcome the gap between research and routine use in daily clinical care.

## Introduction

Neonatal Intensive Care Units (NICUs) play a critical role in the care of premature and critically ill newborns, where meticulous monitoring of vital signs is paramount for ensuring their survival and healthy development. Among these vital signs, heart rate (HR) and oxygen saturations are particularly crucial indicators to tailor medical support and determine the response to intervention. The monitoring of these parameters in this most vulnerable population has always been challenging, due to signal disturbance secondary to motion artefact, compromised circulation [1] and risk of skin lesions especially in those born extremely premature. However, continuous and accurate monitoring of these parameters allows for timely interventions, thereby reducing the risk of complications and improving clinical outcomes [2]. Moreover, the increased need in accuracy and precision of vital signs detection in the newborn lies in the necessity to improve and favour therapeutic measures (i.e., kangaroo mother care), nursing care and imaging evaluations.

Recent advances in technology have significantly enhanced the capabilities of HR and oxygen saturation monitoring in the NICU. Traditional methods, while still the most widespread and used, are being supplemented by innovative approaches that offer greater precision, less invasiveness, and improved comfort for the neonates. These advancements include wearable sensors, wireless monitoring systems, and sophisticated algorithms that provide real-time data analytics and predictive insights. Additionally, vital sign characteristic tools have been shown to be helpful in predicting and detecting episodes of acute clinical deterioration [3, 4] alongside the prediction of clinically relevant long term outcomes [5].

This narrative review aims to explore the future perspectives of HR and oxygen saturation monitoring in the NICU. It will examine current technologies and their limitations, discuss emerging innovations, and consider the potential impact of these developments on neonatal care. In particular, there will be a focus on the development of non-invasive and minimally invasive monitoring technologies, the integration of artificial intelligence and machine learning in monitoring systems, and the implications of these technologies for clinical practice. Through this exploration, we aim to highlight the transformative potential of advanced monitoring systems in enhancing the quality of care in NICUs.

## Aim and search strategies

**Fig. 1:**
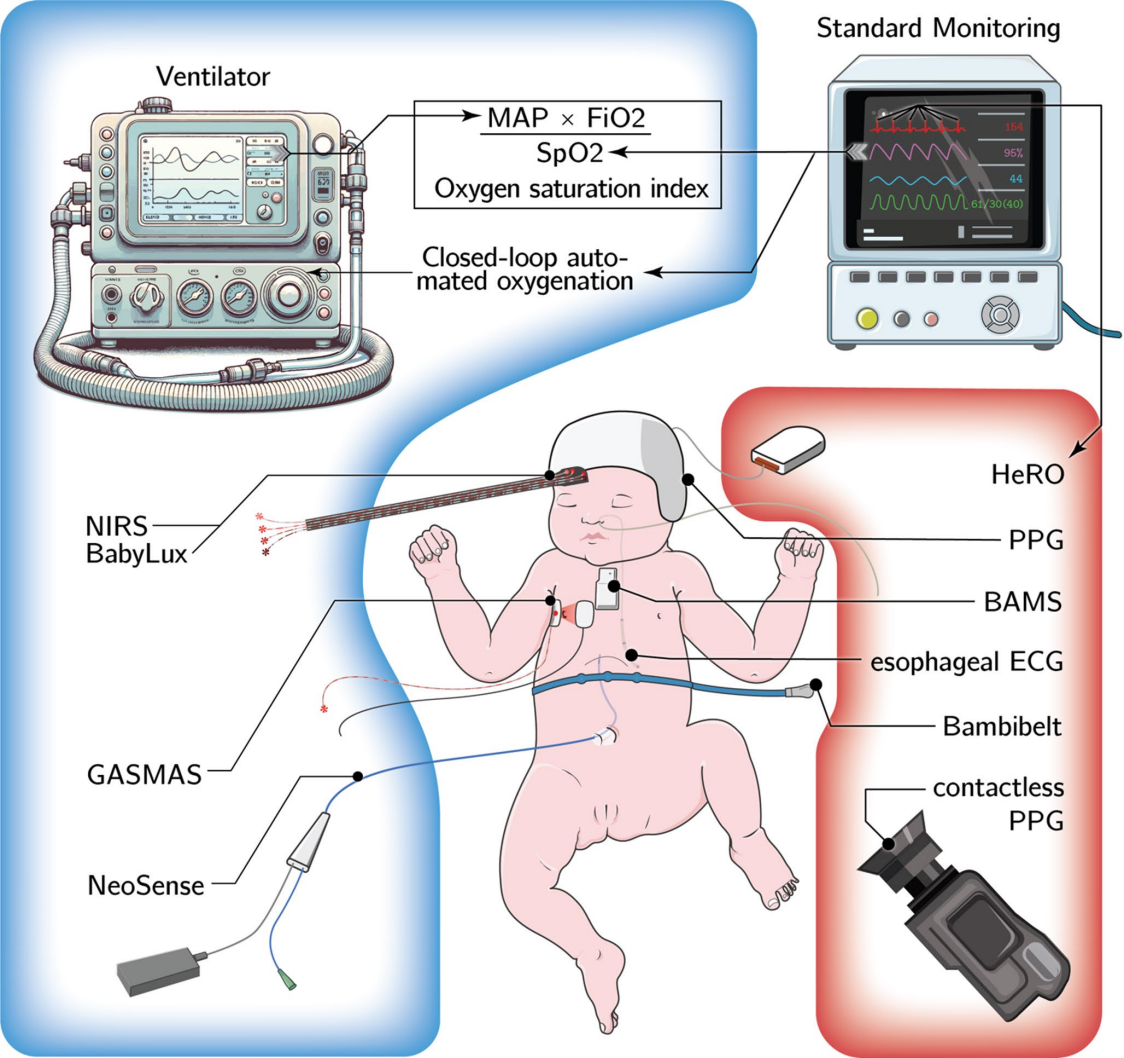
Schematic drawing of monitoring modalities. The monitoring modalities concerning oxygenation are outlined blue, those with heart rate red. Some concepts derive values from traditional monitoring mostly as ECG and SpO_2_ those connections are described by arrows. Surface-based technologies are not depicted in the figure. Abbreviations: BAMS, broadband acousto-mechanical sensing; ECG, electrocardiography; GASMAS, gas in scattering media absorption spectroscopy; HeRO, heart rate observation; MAP, mean airway pressure; FiO_2_, fraction of inspired oxygen; NIRS, near infrared spectroscopy; PPG, photoplethysmography; SpO_2_, pulseoxymetric oxygen saturation. Neonate and Monitor are based on Servier Medical Art under a Creative Commons Attribution 3.0 license

We aimed to describe novel technologies in neonatal monitoring of heart rate and oxygen saturation including secondary data-use - outlining gaps in knowledge, current developments and challenges for future studies. We conducted a narrative review in PubMed, Embase, GoogleScholar, and ClinicalTrials.gov. searching the terms heart rate, monitoring, NICU, oxygenation, preterm infants and technologies. We focused on ongoing or recently published studies, within the last 5 years, including English language only. An overview of discussed modalities can be seen in Fig. 1. Table 1 is summarizing the technical background, main strengths and limitations of the described techniques.

**Table 1:** Overview of different sensing methods, describing used technique, strengths and limitations

Heart rate			
Sensing method	Technical background	Strengths	Limitations
**Photoplethysmography (PPG)**	- Changes in the skin colour due to pulsatile blood flow is detected in the forehead	- Contactless - Commercially available	- Affected by gestational age, birth weight, movements
**Epidermal electronic system (EES)**	- Binodal pair of ultrathin, low-modulus measurement modules.	- Water resistant - Compatible with imaging - High levels of accuracy	- Operating distances - Mechanical fragility
**Transcutaneous electromyography**	- Diaphragmatic activity is wirelessly conveyed from the sensor to the receiver module	- Diaphragmatic activity is wirelessly obtaining both an ECG trace and a respiratory waveform - Commercially available	- Signal contamination by adjacent muscles - Does not classify accurately small diaphragmatic contractions as separate breaths → underestimation of respiratory rate
**Broadband acousto-mechanical sensing** (**BAMS)**	- Continuous acoustic signal from cardiac function and pulmonary airflow	- Wireless, adjunct to current monitoring - Additional information about airflow/ obstruction	- Limited data in infants
**Oesophageal Multichannel-ECG**	- Combination of a gastric feeding tube and a multichannel ECG	- Lack of skin irritation - Less prone to signal artifacts - Commercially available - Additional information about respiration	- Time-consuming postprocessing
**Surface-based technologies**	- Different techniques including among others ballistocardiography; electric potential sensors in mattress - Sensing movement from blood flow (non-contact)	- No sensors needed - Additional information about respiration, movement/ activity state	- Not possible during skin-to-skin contact (patient must lie on special surface) - Movement/ handling artefacts
**Oxygenation**			
**Tremolo sonification**	- Vibrations of the sound relays heart rate, brightness of the sound indicates SpO_2_ levels. - 3 cycles of tremolo indicate M, 6 cycles indicate great deviation from target range	- Eyes-free monitoring - More accurate SpO_2_ ranges during resuscitation	- Absolute SpO_2_ value cannot be judged
**Reflectance pulse oximeters**	- Wireless SpO_2_ probe and data logger on the forehead using photoplethysmographic channels - SpO_2_ estimated from the ratio of red and infrared light	- Less signal interfaces - Less prone to false alarms	- Limited to oxygen saturations of > 85%
**Photoplethysmography (PPG)**	- Forehead tissue perfusion by detecting specific colour changes	- Contactless	- Positive predictive value is low
**Resonance Raman Spectroscopy (RRS)**	- Vibrational spectroscopy to detect oxyhaemoglobin concentration	- Non-invasive tissue oxygenation	- No correlation with SpO_2_
**NeoSense umbilical catheter**	- Intravascular oxygen sensor provides PO_2_ data	- Non-invasive PO_2_ measures	- Clinical study is underway
**Calculated values**			
**Oxygen Reserve Index (ORi)**	- Addition to SpO_2_ - Ranging from 0 to 1 indicating no-reserve to increased-reserve	- Alert prior to a desaturation episode - More precise titration of oxygen	- Clinical testing is still awaited
**Oxygen Saturation Indices (OSI)**	- Surrogate marker of oxygenation index - MAP × FiO_2_ × 100 / SpO_2_	- Mortality prediction with high sensitivity and specificity - Identify ECMO candidates	- Manual calculation needed - Automatic combination of data from different devices is not resolved
**Saturation oxygen pressure index (SOPI)**	- Surrogate marker of alveolar-arterial oxygen gradient - PEEP x FiO_2_/ SpO_2_	- SOPI offers a near-continuous non-invasive assessment tool to monitor the severity of respiratory illness	- Manual calculation needed - Automatic combination of data from different devices is not resolved
**Closed loop automated oxygen systems**	- SpO_2_ values monitored in real time to adjust FiO_2_ without any human intervention.	- Fewer episodes of hypo- and hyperoxaemia - Reduces staff workload - Commercially available	- Mask clinical signs of deterioration - Motion artifacts can mimic hypoxia - No difference in neurodevelopmental outcome
**Techniques using near-infrared lights**			
**Conventional/ continuous wave NIRS (CW-NIRS)**	- Absorption of near- infrared light at different wavelengths enables the calculation of the ratio between oxygenated and deoxygenated haemoglobin	- Potential to improve patient care in several clinical situations - Commercially available	- Measures the changes of concentration and not absolute values of chromophores - Reference value is sensor- dependent and not determined
**Frequency domain NIRS (FD-NIRS)**	- Measures both light intensity attenuation and phase shift, since the intensity of the light is modulated at a particular frequency	- More consistent quantitative (absolute values of haemoglobin) measurements	- Influenced by superficial tissue activity and motion artefacts
**Broadband NIRS**	- Measures the oxidation state of cytochrome-c-oxidase	- Monitor metabolic state at a cellular level	- In vivo measurement is challenging due to small concentration of cytochrome-c-oxidase
**Near Infrared diffuse correlation** **spectroscopy (DCS)**	- Provides an index of cerebral blood flow by quantifying the fluctuations of light scattered by moving red blood cells	- Validated against gold standard methods in adult population - Most sensitive to the motion of blood cells in the microvasculature	- Operation requires some degree of expertise - High- cost - Technical barriers: limited signal-to-noise ratio
**Time-domain NIRS (TD-NIRS)**	- Measures the penetration of ultrashort laser pulses through tissue	- Allows absolute measurement of chromophores	- Complex and costly optical technique
**Combination DCS and time resolved NIRS (BabyLux)**	- Measures blood oxygenation, tissue perfusion and microvascular cerebral blood flow	- Provides a direct measurement of oxygen supply absolute measurement of Hb and HbO_2_ concentrations and blood oxygen saturation.	- Studied in vitro models and in adult population - Trials underway in neonatal settings
**Gas in scattering media absorption spectroscopy (GASMAS)**	- Describes molecular oxygen content within the lungs	- Rapid detection of poor oxygen concentration - Confirms successful intubation, response to surfactant therapy and transition after birth	- Safety profile is not cleared

### Heart rate

Quick and reliable detection of heart rate (HR) in the delivery room can be challenging, especially in infants born extremely preterm. As we enter an era of delayed cord clamping [6] acquisition of an accurate HR immediately after birth may be even more challenging. Heart rate in the first minutes after birth has been used as a predictor of neonatal mortality and subsequent development of brain injury [7, 8], as well as being a reliable indicator of the efficacy of resuscitation provided [9]. The two recommended tools to monitor HR immediately after birth, according to international guidelines [10], are ECG electrodes and pulse oximetry. Although quick in detection and often more reliable than pulse oximetry, ECG electrodes can be difficult to apply properly in a timely fashion and are often not available in many neonatal units as standard equipment, particularly in limited-resource settings. Pulse oximetry on the other hand can be markedly influenced by states of poor peripheral perfusion and motion artefact [11], and although HR values have been shown to be underestimated as compared to formal ECG monitoring this technique can still be utilised to provide useful data even in cases of low perfusion and poor signal quality [11, 12]. This is important as infants transition from the delivery room to the neonatal unit where there is a need for portable monitoring devices which allow for continuous and reliable detection of HR. This review article aimed to focus on newer modalities which have potential to be implemented into clinical care.

#### Non-contact photoplethysmography (PPG)

Lately, several new techniques have been introduced, one of the most studied is photoplethysmography (PPG). The mechanisms underlying PPG are related to contraction and relaxation of the heart, thus determining circulation of blood through the vascular system. Pulsatile blood flow causes subtle changes in the penetration of light through skin and blood vessels which can be detected by infra-red image recording [13]. Indeed, the camera is used as a reflectance imaging photoplethysmography (camera IPPG) by means of algorithmic processing of images, allowing for detection of skin colour changes based on perfusion variations. Intensities vary from red to blue and green, where the green channel represents the cardiac pulse [14]. Several studies [15] have approached these techniques in the delivery room, including both preterm and term infants, although the sample size was limited in most cases. In comparing PPG to ECG, there was overall satisfactory reliability and a good rapidity in HR detection [16]. PPG measurements however may be affected by gestational age, birth weight, heart rate variability, infant movement artefact and skin phototype - thus generalisable clinical reliability is still unclear. Further clinical studies are needed to establish the precision of IPPG-derived heart rate measures in a real-world setting [17].

#### Wireless skin sensors

‘Skin-like’ wireless and battery-free modules for vital sign monitoring, including HR and blood oxygenation, have been tested and proved feasible for use in infants from 28 weeks of gestation through to term. These sensors employ a binodal pair of ultra-thin, low-modulus measurement modules - each referred to as an epidermal electronic system (EES). Such modules are water resistant and compatible with imaging techniques, additionally they have a high mechanical compliance and a non-invasive skin adhesive interface [18], thus making them better suited for the fragile skin of extreme preterm infants. There are, however, some technological barriers to overcome which include the limited operating distances and mechanical fragility of such sensors. Nonetheless, new softer ‘skin-like’ epidermal electronic systems with Bluetooth technology have been tested in pilot studies recruiting fifty patients in both neonatal and paediatric intensive care units, with initial results reporting high levels of accuracy in comparison to hard-wired conventional monitoring systems [19]. An ongoing prospective observational study is assessing the safety and feasibility of this monitoring in a larger cohort of term and preterm neonates (NCT04956354) [20].

The Bambi^®^ belt is a wireless cardiorespiratory monitor that measures the diaphragm’s electrical activity through transcutaneous electromyography (EMG). Transcutaneous electrodes detect the electrical activity of the diaphragm. Diaphragmatic activity is wirelessly conveyed from the sensor to the receiver module, providing HR and respiratory rate (RR) data, with the gating technique removing the cardiac interference from the raw signal thus producing a breathing waveform [21]. A pilot study demonstrated that HR and RR values in preterm infants by means of the Bambi^®^ belt were similar to those obtained through conventional methods such as ECG and chest impedance [22]. A multicentre study is currently in progress which aims to demonstrate non-inferiority of the Bambi^®^belt as an alternative to both ECG and chest impedance monitoring [23].

Broadband acousto-mechanical sensing (BAMS) systems are non-invasive devices which allow for continuous monitoring of respiratory sounds and cardiac cycles [24]. Changes to body sounds can alert clinicians to changes in clinical status and thus act as an adjunct to current monitoring tools. Both high and low frequency sounds are captured within BAMS systems and can quantify cardiorespiratory function and pulmonary airflow during inspiration and expiration. A BAMS device positioned on the suprasternal notch can clearly detect cardiac and respiratory frequency signals and thus provide information regarding airflow within the lung and situations of airflow obstruction [24]. This novel technique has however only been trialled in a handful of infants in the neonatal intensive care unit, and thus further randomised control trials are needed to explore this promising new technique [25].

#### Feeding-tube integrated sensors

The NEO (Neonatal Esophageal Observation) catheter combines a gastric feeding tube with a multichannel ECG. One pilot study has demonstrated high sensitivity and a high positive predictive value for detecting heartbeats with the commercially available Edi catheter (Maquet, Solna, Sweden) and a customised software capturing a multi-channel oesophageal ECG [26]. One advantage of this modality is the avoidance of skin irritation by surface electrodes. The system is also less prone to signal artefacts caused by body movements or interventions, potentially leading to fewer false positive alarms in the NICU [26]. This method has not been fully integrated with real-time implementation into daily clinical practice due to time-consuming post-processing tasks; animal models are however being undertaken to look further into how better acquisition of oesophageal ECG can be delivered in extremely preterm infants, with the aim of developing high-resolution signals with low noise-content.

#### Surface-based technologies

Techniques assessing heart activity from the mechanical stimulus applied on the surface below the patient (e.g. ballistocardiography [27] or textile embedded electrodes [28]) have successfully been studied in newborns as well. Dore et al. for example studied non-contact electric potential sensors embedded in a mattress, on which infants were directly placed after birth. Their preliminary studies showed potential to capture HR within 10 s. The advantage of such techniques lies in not needing adhesives or skin sensors, and in simultaneous measurement of HR, respiratory rate and infant movement [29]. Their strongest limitation besides susceptibility to motion artefacts, might be the fact that the patient does have to lie on the actual sensor, not allowing measurement during skin-to-skin care, which is recommended several hours every day in this patient group [30].

### Oxygen saturation

The utility of non-invasive pulse oximetry within neonatal intensive care has been questioned as to its accuracy in guiding the titration of oxygen delivery [31]. Comparing pulse oximetry values with gold standard arterial blood gas values, using the reference of International Organization for Standardisation (ISO) standards, has enabled the performance of pulse oximetry to be critiqued. One study included 27,237 paired samples from infants with a mean gestational age of 36^+ 6^ weeks [32]. In detecting upper and lower limits of predefined oxygen saturations, pulse oximetry was reported to have fair performance in receiver operating characteristic (ROC) curve analysis, with an area under the curve (AUC) of 0.79. Moreso, internationally binding standards were not fulfilled when oxygen saturations were less than 91%. The high proportion of fetal haemoglobin within extreme preterm infants may however have affected such results because of its effect on the oxyhaemoglobin dissociation curve [33, 34]. The effect of such variables which are known to affect optimal oxyhaemoglobin saturation should therefore be considered when determining adequacy of tissue oxygenation [35]. This study has highlighted the need for determining more accurate and precise measures of oxygen saturation monitoring suitable for the preterm population.

Auditory signals of pulse oximeters (sonification) can allow clinicians to detect changes in oxygen saturation as different pitches of sound are perceived. However, identifying exact changes in the absolute level of peripheral oxygen saturation (SpO_2_) can be hard to determine without observing the visual monitor display. Production of a vibration effect by tremolo modification can therefore be additive to conventional tones when saturations are above or below target range. Indeed, the enhancement of pulse oximetry with tremolo sonification to aid clinician behaviour in determining SpO_2_ during newborn resuscitation has been assessed. Neonatal clinicians determined the SpO_2_ range more accurately (92% versus 62%, *p* < 0.001) when the tremolo sonification device was utilised compared to conventional display [36]. Additionally, the improved accuracy in saturation range identification remained present when clinicians were tasked with an arithmetic distraction problem – suggesting that during neonatal resuscitation when multitasking is required dynamically changing SpO_2_ values will be better identified by clinicians when tremolo sonification devices are utilised.

#### Reflectance pulse oximeters

Standard transmissive pulse oximetry methods use a light source located on the opposite side to a photo-diode, with light passing through the measurement site such as a hand or wrist. Reflectance pulse oximeters have the light source and photo-diode positioned together on the same side [37]. Since these newer reflectance monitors attach to the forehead, they are less susceptible to signal interference secondary to movement artefact [38]. Algorithms have been validated in a newborn dataset with an accuracy of 3.9%, after automatic rejection of 11.7% of the signals with low signal-to-noise ratios as predetermined by a quality index, and thus meet ISO standards [38]. Such validation datasets were however limited to the targeting of oxygen saturations above 85%, and therefore further work within the unstable neonates is required.

#### Non-contact photoplethysmography (PPG)

Utilising digital video cameras to non-invasively measure oxygen saturations by detecting colour changes in the foreheads of infants has been compared to routine methods of pulse oximetry. Such methods of imaging can describe forehead tissue perfusion by detecting specific colour changes when the camera is used as a reflectance photoplethysmograph [17]. The device provided a sensitive measure of desaturation; however, the positive predictive value was low at 20% – likely because the recruited infants were healthy term infants exhibiting an overall low incidence of desaturation episodes. Further clinical studies are warranted to assess the accuracy and precision of infants with unstable physiology.

#### Vibrational spectroscopy

Resonance Raman technology (RRS) uses vibrational spectroscopy to determine non-invasively measured oxyhaemoglobin concentration in the tissues, obtained using a laser and fibre-optic cable and thus producing distinct scattered light wavelengths. Results from a pilot study utilising this technique have not correlated with SpO_2_ values in the neonatal population, with RRS probes (Pendar Technologies, Cambridge, MA, USA) being placed either buccally or on the plantar surface for a ten-minute period. Intra-infant variability of RRS values in those requiring increasing amounts of respiratory support were however reported to be associated with greater instability of the microcirculation despite SpO_2_ being maintained within target range [39]. An observational trial currently being undertaken is assessing whether RRS measures of tissue oxygenation will correlate with standard measures of peripheral oxygenation; and thus, be better at detecting adverse events related to umbilical artery catheter use such as thrombosis, ischaemia, renal failure and necrotising enterocolitis [NCT04038203].

#### Central catheter- integrated sensor

Umbilical arterial catheter monitoring of arterial oxygen tension has been used since the 1970s, allowing for continuous invasive monitoring [40–42]. Continuous monitoring has advantages such as determining the immediate response in arterial oxygenation to changes in respiratory support. Such techniques are however still not widely utilised routinely in neonatal intensive care units with advances in the development of umbilical catheters for such purposes seen over the past years, and improvements in clinical care with regards to reducing thrombotic and infectious events associated with catheter use [43]. A clinical trial is currently underway in Sweden evaluating the reliability of a NeoSense umbilical catheter device in providing oxygen tension data. Besides the usual open lumen for routine medication administration and blood sampling, additions to this customised umbilical arterial catheter include: two closed lumens for pO_2_ electrodes, one silver and one gold connected through two separate wires with the external measuring device, and an additional temperature sensor. Oxygenation values are blinded for the clinical team and compared with blood gas values taken according to routine clinical need [NCT03967587].

#### Spatial oxygenation

Gas in scattering media absorption spectroscopy (GASMAS) is an optical technology which also utilises near-infrared light. This continuous non-invasive modality can act as an adjunct to neonatal respiratory monitoring by describing molecular oxygen content within the lungs. It can provide rapid detection of poor oxygen concentration thus alerting clinicians to pulmonary complications and real-time regional ventilation states [44]. This technology may confer clinical applicability and benefits in confirming successful intubation, assessing response to surfactant therapy and having a role in transition of infants immediately post birth. In a feasibility study of 29 term infants, which included a total of 390 lung measurements, laser spectroscopy was able to clearly detect oxygen gas in 60% of measurements [45]. Furthermore, diode laser spectroscopy tested in newborn piglets identified specific light absorption and transmission patterns in response to lung pathologies of atelectasis and pneumothorax [46]. Long-term safety profile in term and preterm human infants requires evaluation.

#### Near infrared spectroscopy

Optical technologies in neonatal intensive care can provide continuous monitoring of dynamic changes in tissue oxygenation, these techniques are safe and transportable, allowing them to provide bedside monitoring on the NICU. Continuous Wave Near Infrared Spectroscopy (CW - NIRS) is the most common optical technique that calculates the ratio between oxygenated and deoxygenated haemoglobin and displays regional oxygen saturations. Although commercially available, NIRS is sensitive to the balance between local oxygen delivery and oxygen consumption, and distinguishing the contribution of each parameter is often difficult [47]. Moreover, the reference value of regional oxygen saturations is sensor dependent and therefore even routine regular repositioning of sensors can significantly impact its accuracy [48]. These factors limited the routine clinical use of NIRS in neonatal settings despite promising results in several clinical scenarios including: monitoring of fetal-to-neonatal transition [49], treatment of patent ductus arteriosus (PDA) [50], neonatal hypotension [51], identification of infants requiring blood transfusion [52], the detection of neonatal acute kidney injury [53] and monitoring of cerebral autoregulation [54].

One of the most complex and costly optical techniques is time-domain NIRS: this measures the penetration of ultra-short laser pulses through tissue enabling absolute measurement of oxygenated and deoxygenated haemoglobin [55]. The BabyLux device combines diffuse correlation spectroscopy (DCS) with time-resolved NIRS to allow for non-invasive monitoring of cerebral blood oxygenation, tissue perfusion and microvascular cerebral blood flow - and thus can be utilised to provide information on the cerebral metabolic rate of oxygen. This technology has been designed with the preterm population in mind, and recent assessment for accuracy and precision has been determined in both an in vitro model and an adult in vivo study. Stability over several hours of the optical and dynamic properties of the device was reported to be within 3% of the average value, with variability of re-test evaluation of less than 3% [56]. Ongoing clinical trials are assessing performance of the device in newborn infants and preliminary results relating to safety and feasibility are encouraging [57]. The BabyLux device has reported improved trends of cerebral oxygenation and similar blood flow indices in term newborn infants in comparison with other techniques [58].

### Extended use of derived measures

#### Combination indices

The Oxygen Reserve Index (ORI) (Masimo, USA) is a multiwavelength tool used to assess continuous non-invasive pulse oximetry by a mathematical algorithm [59]. This index is measured from 0 to 1 indicating no-reserve to increased-reserve respectively, and thus having the potential to alert clinicians to an impending episode of desaturation in the newborn. This technology has been utilised in one reported case study of an infant undergoing surgical repair for a potential mediastinal leak secondary to tracheo-oesophageal fistula surgery [60]. Such a monitoring tool may aid in guiding the titration of oxygen in newborn infants, in whom precise targeting of peripheral oxygen saturations is required to avoid associated morbidity [2]. A prospective observational study within paediatric anaesthesia recruited 100 infants and found the ORI to provide an early warning for desaturation, however with poor sensitivity and positive predictive values of SpO_2_ [61]. The authors could therefore not recommend routine use of the ORI in its current format in replacing the more widely used SpO_2_ monitoring for impending desaturations in infants.

The oxygen saturation index (OSI) can provide a continuous, non-invasive assessment at the bedside and be utilised to describe efficiency of gas exchange – similar to the oxygenation index, yet the OSI does not require invasive blood gas analysis. The OSI combines values of mean airway pressure (MAP), fraction of inspired oxygen (FiO_2_) and SpO_2_. In one study of infants with congenital diaphragmatic hernia, 49,472 measurements of OSI were analysed. A mean OSI of greater than or equal to 17.3 recorded in the first hour after birth predicted mortality with 100% sensitivity and 91% specificity (AUC 0.94) [62], as well as enabling early detection of those at greatest risk of developing pulmonary hypertension and those requiring postnatal ECMO therapy.

The alveolar-arterial oxygen gradient (A-aDO_2_) is a useful guide for determining causes of hypoxaemia – such as ventilation-to-perfusion mismatch or right-to-left shunting. This however requires invasive arterial blood gas analysis, which is not continuously available. The saturation oxygen pressure index (SOPI) is a continuous non-invasive tool which can enable clinicians in their assessment of infant response to continuous positive airway pressure (CPAP) support. Indeed, SOPI and A-aDO_2_ have been reported to have good correlation (*r* = 0.82), with clinically relevant SOPI values between 1.52 and 1.88 indicating a need for potential escalation of respiratory support with CPAP [63].

Predictive models combining cardiorespiratory physiologic data have been utilised in the preterm population to predict neonatal respiratory outcomes such as oxygen requirement and bronchopulmonary dysplasia. Episodes of periodic breathing, apnoea, intermittent hypoxia and bradycardia were evaluated in a recent study of over 700 infants [64]. A predictive model combining clinical and physiological parameters had an area under the receiver operator characteristic curve of 0.85 on postnatal day of life seven in predicting unfavourable respiratory outcomes. Trajectories of such physiologic parameters can also be trended during the postnatal period and have been shown to vary between infants of different sex and race [65]. Such results may be of benefit to clinicians in tailoring individualisation of care and targeting of specific pharmacologic therapies.

#### Closed-loop systems

Closed loop automated oxygen systems monitor SpO_2_ continuously via transcutaneous saturation probes connected to the ventilator system. Adjustments to the FiO_2_ are made automatically by the pre-defined algorithm within the ventilator so that the infant remains within the predefined target oxygen saturation range. Randomised studies comparing automated closed loop versus manually controlled oxygen administration showed fewer prolonged episodes of hypoxaemia during the automated periods, with greater length of time spent within target oxygen range [66]. Additionally, there was the benefit of a reduced need for manual FiO_2_-adjustments to be made by clinical staff. Longer term clinical relevance of such systems have yet to be determined. One study showed no significant difference in neurodevelopmental outcomes at two years comparing before and after implementation of closed-loop oxygenation within the same NICU [67]. An ongoing randomised multi-centre trial is aiming to assess the effect of automated oxygen control systems on outcomes in extremely preterm infants [NCT03168516] [68].

#### Heart rate characteristics

Physiological changes in the cardiac cycle are initiated in the sinus node and depend on many internal and external factors. These include direct mechanical effects on the heart, a balance between sympathetic and parasympathetic activity, temperature electrolyte concentrations, hypoxia, as well as endocrine substances [69–75]. Heart rate variability (HRV) measures the changes in beat-to-beat duration of the cardiac cycles [76, 77]. In newborn infants, HRV has been used for monitoring response to pain [78–80], describing severity of hypoxic-ischemic encephalopathy [81] and determining physiological maturation in those infants born preterm [82]. Characteristics of vital signs assessed in the time or frequency domain have been proven useful to predict adverse outcomes such as neonatal sepsis [83, 84], duration of respiratory support [85] and extubation failure. Sepsis in preterm neonates can often be associated with decreased HRV even before clinical signs are apparent. This has led to the development of monitors which characterise changes in HRV mathematically and can therefore act as an early warning system to detect those at risk of developing sepsis [86, 87] e.g. the HeRO monitor (Medical Predictive Science Corporation; Charlottesville, Virginia). Indeed, monitoring HRV changes has been shown to reduce septicaemia-related mortality in very low birth weight neonates in the NICU in a large randomised controlled trial (*n* = 3003) [83, 88]. Due to the limited specificity however of HRV monitoring in predicting sepsis [89, 90], there is still scepticism in adopting HRV monitoring routinely into the NICU, for fear of unnecessary administration of antibiotics. Combining HRV monitoring with pro-inflammatory biomarker screening may however counteract this argument by not only aiding in the prevention of unnecessary administration of antibiotics but by supporting clinical decisions regarding antimicrobial therapy [91–93]. Another group proposed using vital signs (heart rate variability and respiratory characteristics) in combination with decreased activity (ECG-derived motion artefacts) preceding neonatal sepsis episodes [94].

Use of HRV to predict other short and long-term neonatal outcomes have also been assessed. One study including more than 600 very low birth weight infants showed a correlation (*r* > 0.93) between cross-correlation of HR and oxygen saturations with adverse outcomes such as late onset sepsis and necrotizing enterocolitis, as well as with the incidence of neonatal apnoea [95]. Furthermore, HR characteristics but not SpO_2_ parameters obtained during an infant’s first days of life have been shown to have an additive predictive effect for the development of cerebral palsy [96]. Vital sign monitoring for the detection of adverse outcomes is dependent however on many contributing factors such as the degree of prematurity, postnatal age at time of measurement and birthweight [84, 97]. This limits the generalizability of such prediction tools to the specific population in which they were validated. Moreso, heart rate variability encompasses a wide range of metrics and thus optimal use and reliability of this vast expanse of data is not utilised to its full potential within neonatal medicine. Trending heart rate variability through the perinatal, postnatal and later paediatric period may in the future allow for precision medicine to be practised routinely, however routine integration of artificial intelligence and machine learning with clinical parameters needs to be better understood and established before this can happen [98].

#### Advanced monitoring data analysis

**Fig. 2:**
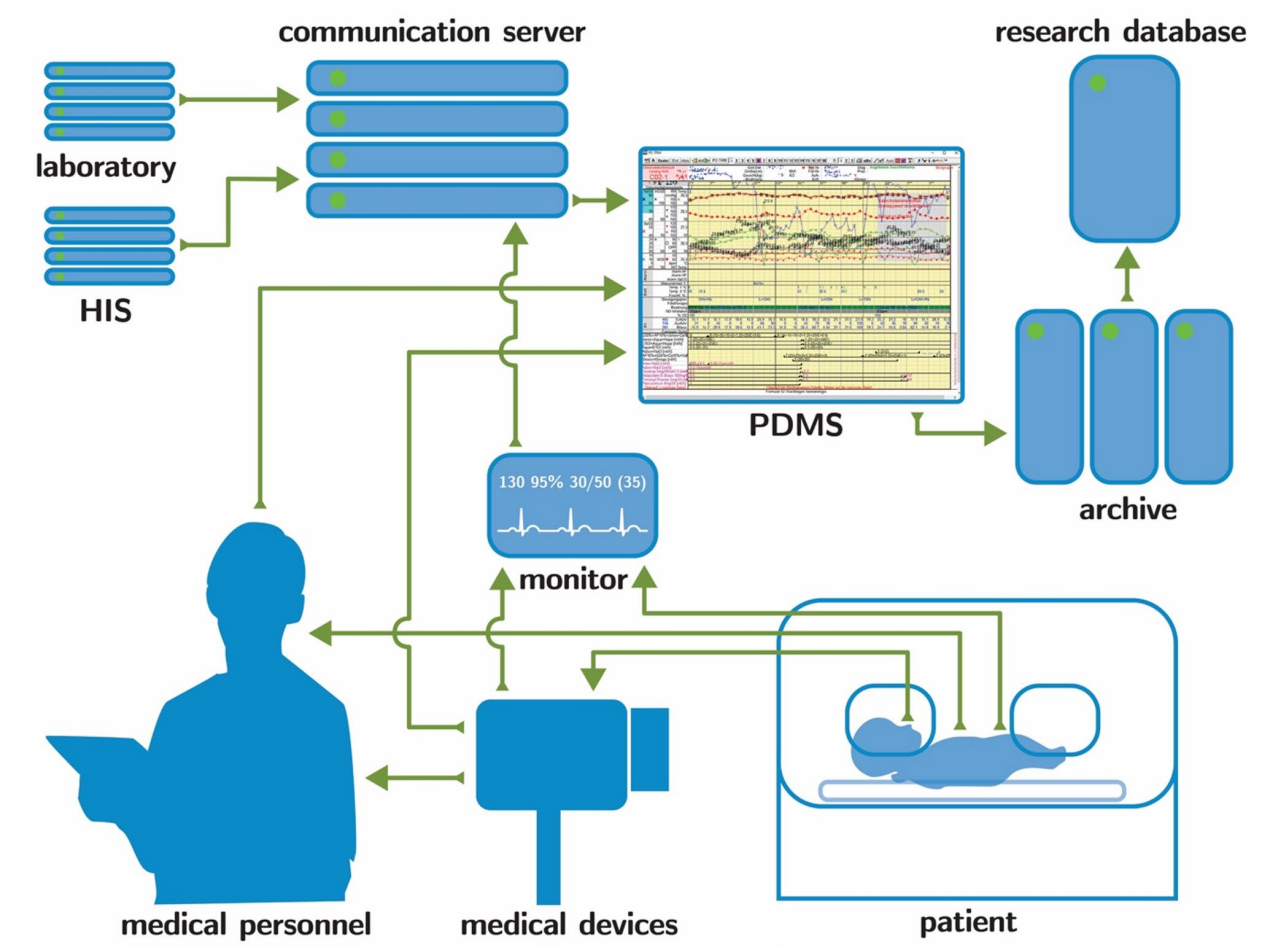
Data flow

Continuous monitoring techniques can provide an immense number of data points at frequent time intervals. The capture and storage of such data can enhance the opportunities for subsequent longitudinal secondary data analysis [99]. Data flow in NICU has many interactions - as depicted in Fig. 2. If patient data management systems (PDMS) can be readily accessible, they could enable advancement of research by allowing for interpretation of large datasets of routinely recorded clinical inputs. Challenges to routine data collection, analysis and storage of some of the new modalities presented above are in part because of their early stage in development. They are often error-prone, and ownership rights can make them difficult to standardise with telemetry and PDMS solutions. Combining current monitoring techniques of already verified existing modalities may act as a bridge until new modalities are readily available.

Flowchart of data streams from the neonatal intensive care unit from the patient into research. Abbreviations: HIS, hospital information system; PDMS, patient data management system.

### Current and future challenges

Improvement of heart rate and oxygenation monitoring techniques in critically ill neonates is a fast-growing research area. Some of the novel monitoring modalities described will supersede one or more of the current ones currently available. At present, many of the techniques described in this article are promising, but currently not implemented in daily clinical care. The dominant reasons for lack of implementation include, but are not limited to, the following described categories.

#### Clinical utility and relevance

Different techniques have so far only been applied in very controlled study settings, with many not tested under clinically meaningful situations such as in the delivery room or in the NICU. Both safety and feasibility under clinical circumstances are crucial when it comes to vital sign monitoring and these newer techniques must be tested in large prospective studies comparing them with current standard methods of monitoring in non-inferiority trials. This is of particular importance within the intensive care setting where monitoring tools are utilised to detect potential drops in heart rate and oxygenation, whilst at the same time minimising false alarms. Furthermore, in critical care environments using novel camera devices may not be the most suitable modality of choice. Indeed, the camera needs access to the infant as well as clinicians requiring full view of the camera screen. Primary viewing of the video screen therefore has the potential to be blocked by both caregivers and other equipment which can thus render them impractical and ineffective in cases of sudden cardiopulmonary instability where accurate signal registration is important. Such monitoring techniques may therefore not be the modality of choice in NICU and may be more suited to lower intensity environments. Additionally, many tools relying on surface sensors are prone to signal artefact and signal saturation through muscular activity or handling of the patient [21, 22], and therefore similar to currently utilised peripheral oxygen sensors might be susceptible to episodes of inaccuracy due to loss of signal. There is however a pressing need for such non-invasive monitoring tools within the fragile preterm population – indeed currently utilised transcutaneous monitors often need to be frequently re-sited to avoid skin breakdown and damage [100].

#### Costs, feasibility and challenges of introducing novel technologies

Advances in monitoring techniques for both heart rate and oxygen saturations may prove costly both in terms of money for expensive material, mandatory licencing for data acquisition programs [14, 26, 101] and where complex training is required for staff – although over time, staff workload is expected to reduce as has been shown in automated closed loop oxygen control [102]. Multi-disciplinary team input is necessary to ensure confidence and empowerment of appropriate interpretation of non-invasive monitoring techniques by the clinical team, thus allowing staff to focus on other important areas of care [66]. Furthermore, medical equipment can pose difficulties to family integrated care – with multiple wires and noises from machines being reported by parents as being intimidating and posing a barrier to kangaroo care [103]. This is an important aspect of care which needs to be considered when developing new technologies and monitoring devices. Another form of ‘cost’ is time, in terms of extensive signal cleaning or post-processing steps, hindering immediate and routine implementation into clinical practice [26]. Additionally, lack of consistency between different studies leads to insufficient concrete evidence to implement tools such as NIRS. Underlying reasons for conflicting results can be due to the characteristics in observed time points, patient populations, clinically relevant outcomes, and guideline differences between institutions.

#### Challenges of clinical decision support tools, using vital sign characteristics

The final aim of any new monitoring technique or tool should always serve to improve patient outcomes. This may be achieved through more accurate and reliable measurements or through provision of additional information to support staff in clinical decision making. However, in an era promising a future of big data and machine learning, there needs to be proper evaluation of such techniques, with investigators having easier access to more standardised data. A common hindrance to the implementation of clinical decision support systems (CDDS) into routine practice is the interruption of work-flow or extra-time required from clinicians to manually feed in information in a separate tool or system [104]. Additionally, many CDSS are prone to false alarms leading to alarm fatigue and distrust [104]. Machine learning algorithms are usually trained on a subpopulation with specific biological behaviour; however, this can lead to overfitting of data and therefore subsequent results cannot be generalised when applied to a different, more heterogeneous population. This is of special importance in the neonatal population, where the physiology of extremely preterm infants is hugely different to those born at full term with perinatal complications or congenital malformations. Moreover, it can be difficult to convince an experienced clinician to utilise a new system or toolkit. It is therefore important that such supporting systems are understandable and interpretable. For example – in predicting neonatal sepsis by interpretation of heart rate characteristics, if clinicians do not understand how the alarming vital sign behaviour is to be understood, the still negative (low) value of biomarkers could be wrongly reassuring.

#### Data framework

Health data is sensitive and analyses therefore are strictly regulated. It is also important to consider regulatory aspects in addition to clinical utility, safety, and feasibility. Medical devices in the United States are required to be approved by the FDA, and heterogeneity of the legislative framework as assessed by the European Union can also be problematic with obstacles relating to data protection and security. Such legislation can prove to be especially challenging when designing projects spanning multiple countries. There are however universal rules regarding anonymization and pseudonymization as governed by laws under General Data Protection Regulation (GDPR) [105]. The collaboration of NICUs becomes more important in data driven neonatology: with small units needing to work together to gather data, the acquired data being used beyond real-time monitoring and support of clinical decisions, with subsequent integration of this data with other sources. Harmonisation to facilitate data-sharing therefore remains highly important in view of the collaboration between NICUs that is necessary to gather the amount of data required for large, randomised controlled trials and the subsequent training of artificial intelligence.

We acknowledge that given the nature of this review (not systematic), the overview of displayed techniques might not be complete. Additionally, we were not able to do a performance comparison between established and newly developed monitoring techniques. This would be of utmost importance for studies developing new technologies.

## Conclusion

New methods for monitoring and predicting heart rate and oxygenation in newborns are currently in development. Ensuring these technologies are user-friendly, easy to understand, and safe is crucial. However, there are significant challenges, including high costs, complex study designs for extremely premature infants, and the need for extensive validation. Also, the predictive capabilities of these technologies could greatly improve outcomes by enabling early detection and timely interventions. Continued vigilance and research are essential.

## Data Availability

No datasets were generated or analysed during the current study.
